# Fulminant Hepatic Failure and Fatal Cerebral Edema Following Clostridium perfringens Bacteremia: Case Report and Review of Literature

**DOI:** 10.7759/cureus.1714

**Published:** 2017-09-26

**Authors:** Alice Shen, Gabriel O Ologun, Robert Behm

**Affiliations:** 1 General Surgery, Guthrie Clinic/Robert Packer Hospital; 2 Trauma/Critical Care, Guthrie Clinic/Robert Packer Hospital

**Keywords:** clostridium perfringens, fulminant liver failure, hepatic abscesses, fatal cerebral edema, diabetes mellitus

## Abstract

Clostridium perfringens (CP) bacteremia is a rare but rapidly fatal infection. Only 36 cases of CP bacteremia with gas containing liver abscesses on image studies have been reported in the literature since 1990. In this report, we describe a 65-year-old diabetic male with CP bacteremia which progressed into fulminant hepatic failure with subsequent fatal cerebral edema.

## Introduction

Clostridium perfringens (CP) bacteremia has been described as a rare and rapidly fatal disease secondary to massive hemolytic anemia, resulting in progressive multi-organ failure. The mortality rate exceeds 80% despite high-dose intravenous antibiotics and source control [1]. It is commonly associated with diabetes, malignancy, and immunosuppression [2]. In this report, we present a 65-year-old diabetic male with CP bacteremia which progressed into fulminant hepatic failure with subsequent fatal cerebral edema.

## Case presentation

A 65-year-old male with a history of diabetes mellitus and coronary artery disease presented to an outside hospital emergency department with an one-day history of abdominal pain. He was febrile to 101.3 °F with leukocytosis of 32K/µL, elevated transaminases and hyperbilirubinemia (total bilirubin of 7.8 mg/dL). The working diagnosis was cholangitis. The patient was started on broad-spectrum intravenous antibiotics, and transferred to our hospital for endoscopic retrograde cholangiopancreatogram (ERCP) and further management.

**Figure 1:**
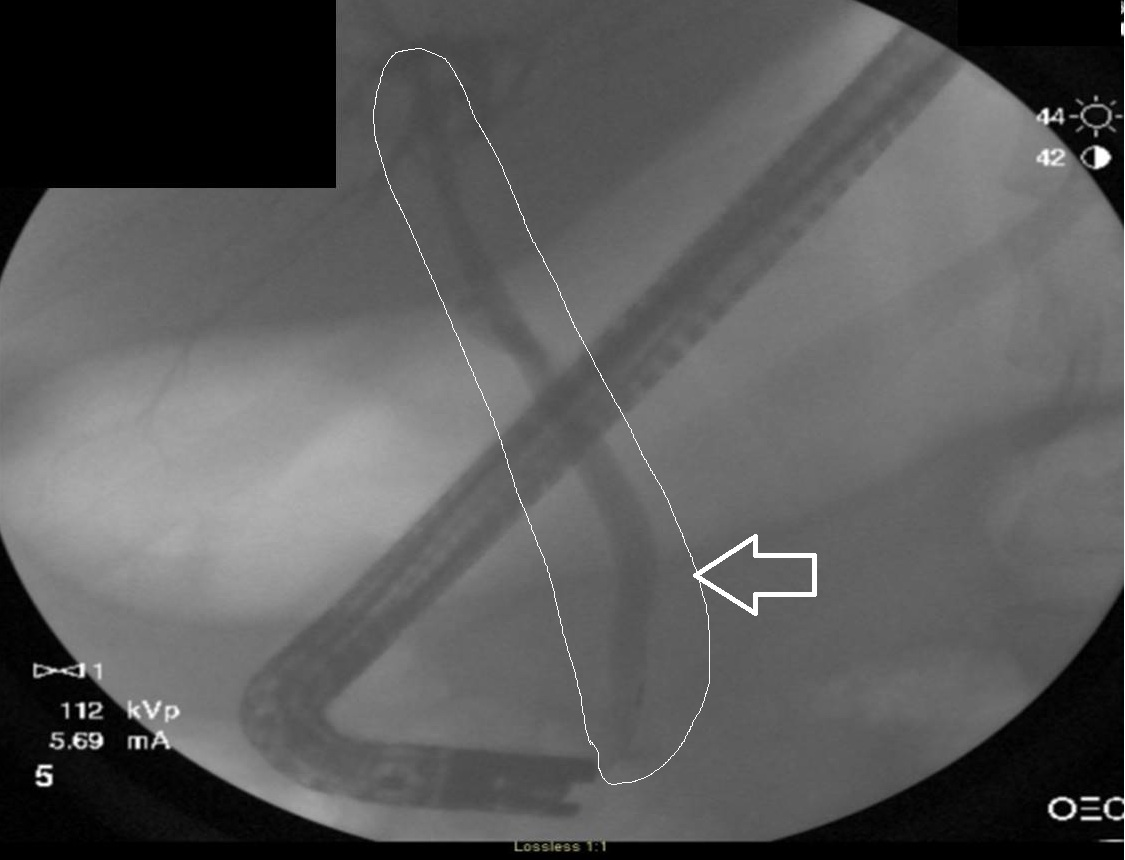
The first endoscopic retrograde cholangiopancreatogram (ERCP) revealing absence of filling defect in the biliary tree (outlined area and arrow).

**Figure 2:**
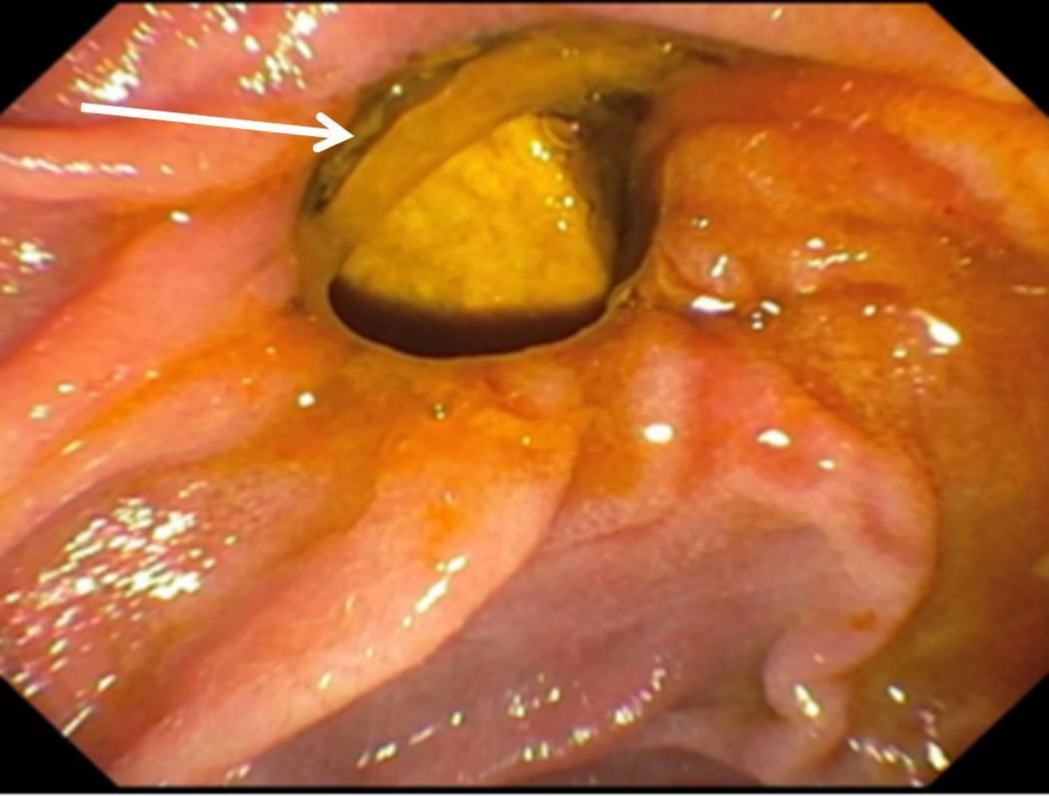
Duodenal diverticulum containing stone.

**Figure 3:**
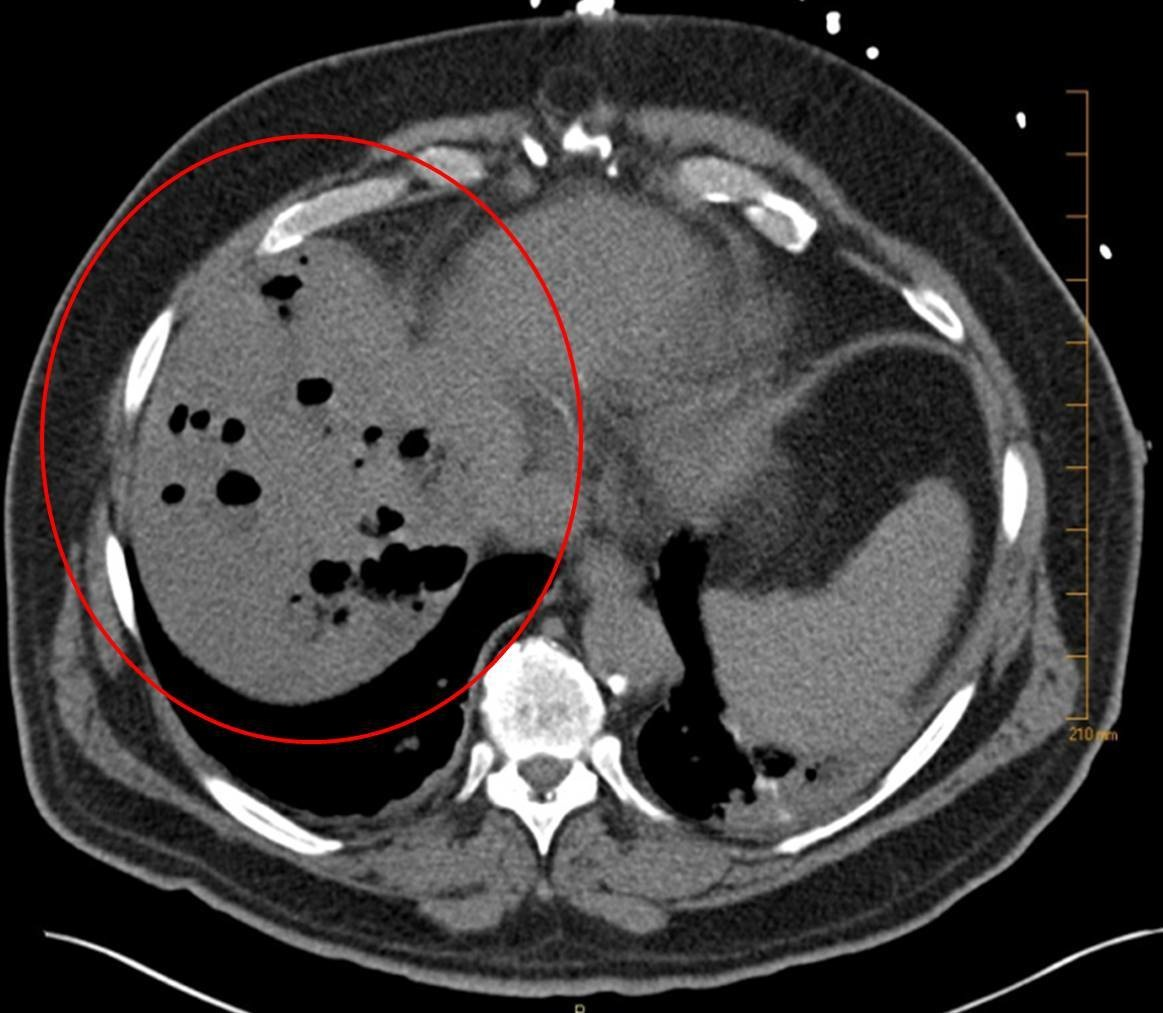
Multiple hepatic abscesses and multiple air fluid levels.

The ERCP revealed no evidence of biliary duct obstruction (Figure 1), and a non-obstructing biliary stone was found in a duodenal diverticulum associated with the major papilla (Figure 2). A computed tomography (CT) scan of the abdomen and pelvis was obtained which showed multiple hepatic abscesses containing air fluid levels involving the right hepatic lobe. These abscesses were not amenable to percutaneous drainage (Figure 3). Due to concerns of gaseous organism infection, repeat ERCP with sphincterotomy and stent placement were performed with the intention of source control. Blood culture from the outside hospital returned as Clostridium perfringens.

**Figure 4:**
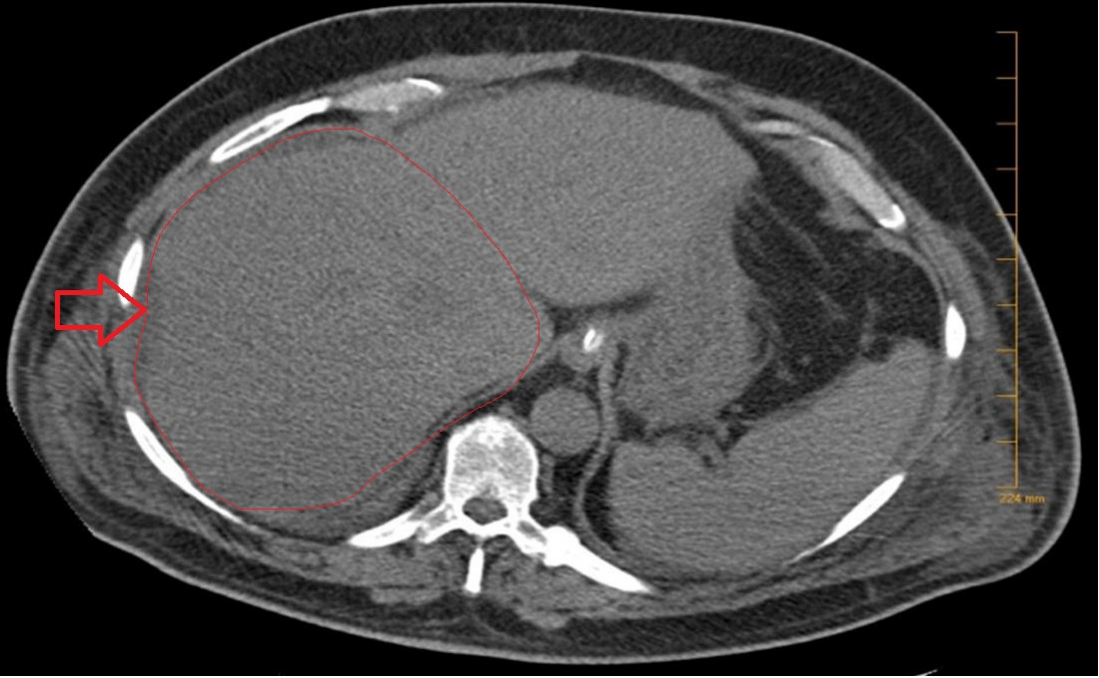
Resolution of liver abscess status post-surgical drainage. Outline and arrow indicating area of interest.

On hospital day four, general surgery was consulted. Repeat CT scans of the abdomen showed no worsening of the liver abscesses. However, the patient continued to decline clinically, with sepsis progressing to multi-system organ failure, requiring hemodialysis. Furthermore, he developed an altered mental status and was intubated for airway protection. Given his worsening clinical picture, the patient was taken to the operating room where he underwent open surgical debridement and drainage with ultrasound guidance. The CT scan obtained on postoperative day two demonstrated resolution of liver abscesses (Figure 4). The blood cultures obtained in our institution were negative. However, the hepatic abscess culture grew Saccharomyces cerevisiae (SC) after 10 days of incubation.

**Figure 5:**
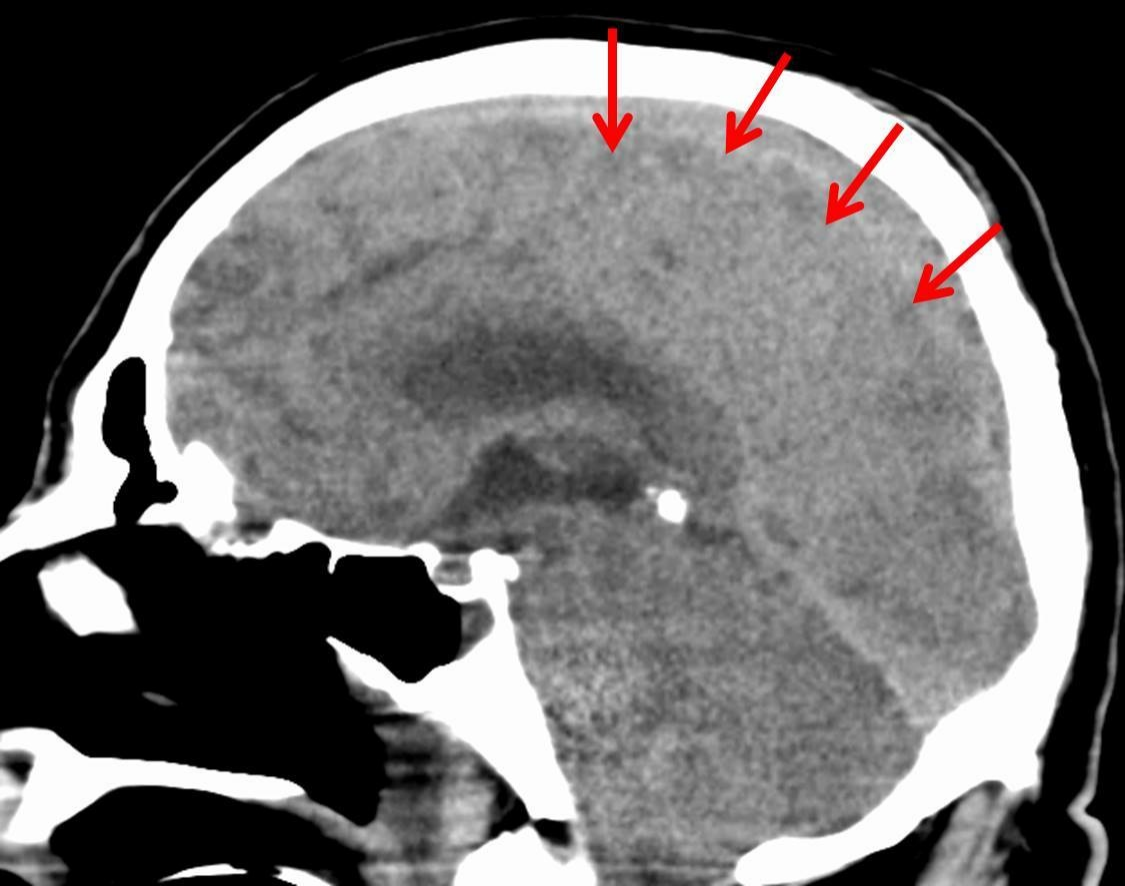
Severe and diffuse cerebral edema with obstructive hydrocephalus.

On postoperative day three, the patient had a seizure episode, prompting a CT scan of the head which demonstrated cerebral edema. Magnetic resonance imaging (MRI-angiogram) of the head was done, revealing no vascular abnormality. The cerebral edema was thought to be secondary to acute liver failure. A repeat CT scan of the head, on postoperative day five revealed progressive diffuse cerebral edema with the development of obstructive hydrocephalus (Figure 5). Neurosurgery was consulted, who felt further intervention would not alleviate an impending herniation. On hospital day eleven, the patient's family opted for comfort care measures. He was terminally extubated, expiring shortly thereafter.

## Discussion

Law and Lee [3] reviewed 20 cases of CP bacteremia with liver abscesses from 1990 to 2012, showing a median time from admission to death of 11 hours. In contrast to those described in the literature, our patient did not have massive intravascular hemolysis, hypotension on presentation, or a rapidly fatal outcome. Although his initial blood cultures were positive for CP at outside hospital, his repeat blood cultures in our hospital were negative for organisms. This negative result could be explained by early initiation of intravenous antibiotics.

The patient’s hepatic culture data was significant for the late growth of Saccharomyces cerevisiae. One case of liver abscess positive for SC was described in 1990 [4]. In this review, CP was also isolated in the liver abscess culture but the blood culture was positive for Pseudomonas aeruginosa. This discordance of microorganism between blood culture and liver abscess cultures was also described in other cases of CP abscess [5-6].

It is unclear whether this patient truly had CP liver abscess because hepatic fluid failed to isolate CP. Nevertheless, he presented with clinical features of pyogenic liver abscess: fever, epigastric pain, bacteremia, and the presence of gas on liver image studies. The liver culture could be false negative for CP. There was also a case in the literature reported with positive CP blood culture with failure to isolate CP from liver abscess culture [7].

We proposed that his fulminant hepatic failure was the primary event leading to his clinical outcome. This would explain the lack of CP in the liver abscess culture. The majority of fulminant liver failure are idiopathic. The hepatic cell death and necrosis, as well as the ERCP performed prior to the CT scan, could contribute to the hepatic gas shown on the CT image. The abscess formation pattern during the debridement was noted to be in the hepatic vein distribution, which has been shown to be more consistent with liver necrosis rather than a liver abscess. This could explain the patient’s lack of overwhelming shock expected with CP bacteremia. To our knowledge, this is the first documented case of CP bacteremia associated with fulminant liver failure which progressed to fatal cerebral edema.

We reviewed 37 cases of CP bacteremia with associated liver abscesses reported since 1990 (Table 1). These cases had an average age of 67 years (range: 42 to 84; 26 patients were male (70%), and 11 were female (30%). The most common underlying condition was diabetes (41%). There were eight patients with diabetes as the only medical co-morbidity. Ten patients (27%) had malignancy. Two (5%) had a history of transplantation for primary sclerosing cholangitis or alcohol cirrhosis who were on immunosuppressive therapy. Eight (20%) had a history of hepatobiliary pancreatic operations or procedures within two months prior the diagnosis of CP liver abscesses. Six (16%) had no underlying conditions.

**Table 1:** Cases of Clostridium perfringens infection associated with gas-containing liver abscesses on image studies published since 1990. Hb = reported hemoglobin (g/dL); WBC = white blood cell count (K-g/L); TBili = total bilirubin (mg/dL); LDH = Lactate dehydrogenase; M = male; F = female; DM = diabetes mellitus; CBD = common bile duct; CA = cancer; TACE = transarterial chemoembolization; ESRD = end-stage renal disease; HD = hemodialysis; HCC = hepatocellular carcinoma; RY HJ = Roux-en-Y hepatojejunostomy; MW = microwave; Yes-p = percutaneous drainage; Yes- O = operative drainage; BCx = blood culture; Liver Cx= Liver Culture; + = positive for Clostridium perfringens; nr = not reported or not obtained; E. coli = positive for E. coli but negative for Clostridium perfringens; – = negative for C. perfringens; (a) = autopsy; S.c.= positive for Saccharomyces cerevisiae, negative for Clostridium perfringens. Hrs = hours; N/A = does not apply. References mentioned in this table are available on request.

No.	Author	Year	Age	Sex	Underlying Conditions(s)	WBC	Hb	TBili	BCx	Hepatic drain	Liver Cx	Survi-val	Hrs present. to death
1	Batge [7]	1992	61	M	DM, pancreatic cancer s/p Whipple	38.2	11.6	43.9	+	Yes – p	–	Yes	N/A
2	Rogstad	1993	61	M	None	N/A	16.7	N/A	+	No	+ (a)	No	3
3	Gutierrez	1995	74	M	None	19.8	13.1	4.1	+	No	+ (a)	No	6
4	Jones	1996	66	F	Liver Transplant	11.3	11.3	4.4	+	No	+ (a)	No	10
5	Eckel	2000	65	F	CBD CA meta s/p TACE	13	11.2	4.6	+	Yes – p	nr	Yes	N/A
6	Kreidl	2002	80	M	DM, ESRD on HD	29	10.7	12.6	+	No	+ (a)	No	11
7	Pichon	2003	42	F	Alcohol cirrhosis	6.2	10.2	12.3	+	No	nr	Yes	N/A
8	Quigley	2003	73	M	Ischemic heart	11	14.2	4.2	nr	No	+ (a)	No	Died at home
9	Bergert [5]	2004	58	M	Pancreatic cancer s/p Whipple 1 yr	34.1	N/A	7.7	E. coli	Yes – p	+	No	24
10	Au	2005	65	M	DM, ESRD on HD	25	6.2	9.4	nr	No	+	No	72
11	Fondran	2005	63	M	pancreatic cancer meta to liver	N/A	N/A	N/A	+	Yes – O	nr	Yes	N/A
12	Daly	2006	80	M	DM	N/A	8.7	N/A	+	No	nr	No	3
13	Ohfani	2006	78	M	DM	18.6	10	1.4	+	No	+ (a)	No	3
14	Loran	2006	69	M	None	26	8.7	N/A	nr	No	+ (a)	No	6
15	Alarcon Del Agua	2009	74	M	Stroke	16	N/A	3.7	+	Yes – O	+	Yes	N/A
16	Merino	2009	83	F	None	26.5	12.2	19.6	+	No	nr	No	72
17	Meyns	2009	64	M	DM, myelodysplastic syndrome	1.5	7.2	8.3	+	Yes – p	+	No	48
18	Tabarelli	2009	65	F	pancreatic cancer s/p Whipple	N/A	N/A	N/A	+	Yes – p	+	No	120
19	Bradly [2]	2010	52	M	HCC s/p RY HJ	11.6	N/A	17.4	+	No	+ (a)	No	6
20	Ng	2010	61	F	DM	16.8	13.5	15.4	+	Yes – O	nr	Yes	N/A
21	Rajendran	2010	58	M	None	14.6	13.3	N/A	+	Yes – O	+	Yes	N/A
22	Law [3]	2012	50	F	Rectal Cancer	46.3	8.3	8.9	+	Yes – p	nr	No	168
23	Qandeel	2012	59	F	DM, s/p cholecystectomy	N/A	9.1	N/A	+	Yes – O	nr	Yes	N/A
24	Sathiyam-oorthy	2012	65	F	DM	N/A	N/A	N/A	+	Yes – p	nr	Yes	N/A
25	Imai	2014	76	M	HTN	33.2	12.2	10.5	+	Yes – p	+	No	5
26	Kitterer	2014	71	M	Liver Transplant	18.5	11.2	3.7	+	Yes – O	nr	No	13
27	Kurasawa	2014	65	M	DM	24.8	13.5	6.4	+	No	nr	No	6
28	Cochrane	2015	65	F	DM	normal	13	9.6	+	Yes – p	nr	Yes	N/A
29	Eltawansy	2015	81	F	Stroke	22	N/A	0.4	+	Yes – p	+	No	120
30	Khan	2015	77	M	DM, HTN	33	6	9.5	–	No	+ (a)	No	7
31	Li	2015	71	M	HCC s/p TACE	11.5	11.8	18.9	+	No	nr	Yes	N/A
32	Rives	2015	63	M	Colon CA meta to liver. ERCP for obs. Jaundice	21.3	N/A	7.6	+	Yes – p	nr	Yes	N/A
33	Garcia	2016	65	M	DM, Chiari I	22.2	14.6	2.6	+	Yes – p	+	Yes	N/A
34	Hashiba	2016	82	M	DM	30.1	8.3	9	+	No	nr	No	2
35	Kyang [6]	2016	84	M	gastric cancer meta to liver s/p MW ablasion	4	N/A	N/A	E. coli	Yes – p	+	Yes	N/A
36	Lim	2016	58	M	None	14.2	12.4	N/A	+	No	nr	No	7.5
37	Shen	2017	65	M	DM	32	12	7.8	+	Yes – O	S. c.	No	264

The relationship of the blood and liver abscess culture results was also evaluated. CP infection was diagnosed on the basis of positive blood or liver abscess culture results, although only 13 cases (35%) had definitive concordance between the two culture results. Nine cases (25%) of positive liver abscess cultures were from the autopsy. Fifteen (41%) cases did not report or did not obtain liver abscess culture results.

The survival rate is only 37.8% among the reviewed cases. Of the 14 survivors, 12 (85%) had the liver abscesses removed. The median time from presentation to death for mortality is 10.3 hours, which is similar to the previous report of 11 hours [3].

## Conclusions

CP bacteremia remains a rare but life-threatening disease which requires timely diagnosis, early systemic antibiotics, and source control. A timely diagnosis could be a challenge because of the time required for the blood culture result. A clinical picture of fever, epigastric pain, bacteremia, and hepatic gas-formation on imaging studies should prompt the diagnosis of CP infection. For associated liver abscesses, either percutaneous drainage or surgical drainage is acceptable. For patients who present with acute liver failure, timely referral to a transplant center is imperative to improve the chance of survival.
